# Development and validation of the creative personality and thinking styles scale: a multifaceted measure for assessing creativity-related individual differences

**DOI:** 10.1186/s40359-026-04959-8

**Published:** 2026-06-08

**Authors:** Masahiro Yoshida

**Affiliations:** Faculty of Nursing, Musashino University, 3-3-3 Ariake, Koto-ku, Tokyo, 135-8181 Japan

**Keywords:** Creativity, Creative personality, Creative thinking style, Creative Personality and Thinking Styles Scale

## Abstract

**Background:**

Creativity is the process of generating contextually useful novel outcomes by uniquely reconfiguring existing knowledge and information. While various measures examine specific aspects of creativity, multifaceted measures that holistically assess creativity as a domain-general factor are still needed. This study developed the Creative Personality and Thinking Styles Scale (C-PETS), which measures personality, thinking styles, and creativity-related motivation based on Plucker et al.’s theory of creativity.

**Methods:**

The initial C-PETS item pool was developed by referencing existing scales, followed by a qualitative screening process to ensure theoretical consistency. Exploratory factor analysis involving 572 participants was conducted to examine the underlying factor structure. Subsequently, confirmatory factor analysis and a higher-order model analysis involving 943 participants were performed to verify the model fit.

**Results:**

Exploratory factor analysis identified a five-factor, 16-item scale comprising Broad Thinking, Thorough Thinking, Information Manipulation, Challenge-Seeking, and Ambiguity Tolerance. The higher-order CFA model demonstrated an excellent fit (GFI = .97, CFI = .98, RMSEA = .02). Criterion-related validity was supported by significant correlations with the Short Scale of Creative Self-Efficacy (*r* = .75), Aesthetic Experiences Scale (*r* = .30), and Divergent Association Task (*r* = .18). Test–retest reliability after two weeks was strong (*r* = .87). Additionally, a positive correlation was observed with educational attainment.

**Conclusions:**

The C-PETS represents a valuable contribution to creativity assessment by providing a multifaceted measure of creativity-related personality, thinking styles, and motivation.

**Supplementary Information:**

The online version contains supplementary material available at 10.1186/s40359-026-04959-8.

## Background

Creativity is defined as the interaction among aptitude, process, and environment by which an individual or group produces a novel and useful product within a social context [1]. Plucker et al. [1] defined aptitude as comprising domain-general personal traits (personality, cognitive styles, and motivation) and domain-specific factors (expertise), emphasizing the importance of multifaceted investigations into domain-general individual difference factors. Furthermore, they identified “process” as consisting of divergent thinking as well as the evaluation and refinement of ideas.

Historically, creativity research has centered on divergent thinking—the ability to generate a diverse range of solutions [2]. In response, Plucker et al. criticized the field’s disproportionate focus on process-oriented research [1]. Moreover, evidence suggests that the correlation between divergent thinking tests and creative achievement is small, indicating that divergent thinking has limited explanatory power regarding creative outcomes [3]. However, the definition of creativity as an interaction between aptitude, process, and environment has also faced criticism; its measurement is complex, and which factors are primary or how these theoretical components are interrelated remains unclear.

To address these issues, this study aims to develop a multifaceted tool that integrates multiple psychological factors, including personality, thinking styles, and motivation into a single framework, and analyze individual differences such as age, gender and educational attainment.

### The dominance of divergent thinking and its limitations

Modern creativity research is widely considered to have been catalyzed by Guilford’s presidential address to the American Psychological Association, in which he emphasized the importance of studying creativity [4]. Guilford was the first to address divergent thinking in creativity research. He proposed the Structure of Intellect model, distinguishing divergent thinking from convergent thinking [5]. Divergent thinking is a cognitive process that generates diverse solutions for a single problem. Guilford considered divergent thinking to be the cognitive function underpinning creativity. A widely-used representative tool for assessing this construct is the Torrance Tests of Creative Thinking (TTCT). The TTCT measures divergent thinking using four indicators: fluency, flexibility, originality, and elaboration [6]. Other tests measuring divergent thinking include the Alternative Uses Test [7], which asks participants to list as many novel uses as possible for everyday objects, and the Remote Associates Test, which requires participants to identify a common word linking a series of unrelated words [5]. While these divergent thinking tests have been widely used as indicators capturing one aspect of creative potential, they do not comprehensively measure creativity [8].

### Multifaceted theories and the need for an integrative measure

Against this background, research has emerged that seeks to conceptualize creativity from a more multifaceted perspective. Amabile [9] proposed a component model of creativity, arguing that three elements—domain-relevant skills, creativity-relevant processes, and intrinsic motivation—are crucial, particularly positioning intrinsic motivation as the central element for creative outcomes. Investment theory also adopts this multifaceted approach to creativity [10]. Sternberg and Lubart proposed that six resources shape creativity: intellectual ability, knowledge, personality, thinking style, motivation, and environment [10]. However, theories which aim to understand creativity from multiple angles, such as those by Amabile, Sternberg and Lubart, and Plucker, face the criticism that theories explaining creativity are highly diverse. It remains unclear which factors are primary and how these theories interconnect [11]. Furthermore, few studies treat creativity as decomposable, with operationally testable empirical elements, making causal verification difficult. However, as empirical research continues to uncover new individual facets of creativity, the need for multifaceted assessments that integrate these diverse components is becoming increasingly pressing.

### Personality, thinking styles, and motivation as core individual differences

While critiques of research centered on divergent thinking and the recognition of the importance of multifaceted approaches have grown, the complexity involved in measuring such a comprehensive construct has frequently been noted. Nevertheless, substantial progress has been made in investigating its individual components. Regarding the personality of creative individuals, it has been suggested that they tend to seek new challenges [12], that creativity is more likely to emerge from individuals who set challenging goals [13], and that creative individuals exhibit significantly higher tolerance for ambiguity [14] compared with non-creative individuals. Ambiguity tolerance—the tendency to not find ambiguous situations unpleasant but rather to enjoy them—has been noted as fertile ground for generating original ideas [12]. Regarding aptitude, it is related not only to the aforementioned items but also to divergent thinking [1, 15].

Other personality and thinking styles linked to creativity include openness to experience [10] and absorption [16]. Adaptive perfectionism, which involves setting high goals for oneself, has also been linked to creativity owing to the tendency to persist in the face of difficulties and setbacks [17]. Furthermore, metacognition—the ability to reflect on one’s own cognitive processes—is associated with creativity, particularly divergent thinking [18, 19] and it is thought to function by selecting superior ideas and discarding inferior ones [20], specialized curiosity (focused curiosity aimed at filling specific knowledge gaps), and diffuse curiosity (exploratory curiosity seeking novelty and diversity) [21]. Additionally, the need for cognition—a tendency to actively engage in and enjoy thinking and intellectual activities related to motivation—is associated with creativity, particularly divergent thinking [22]. Indeed, these studies examined the relationship between creativity and individual personality or thinking styles.

In the 2000s, advances in neuroscience enabled the measurement of the default mode network. This revealed that during mind-wandering—a state where conscious attention drifts from “here-and-now” tasks toward internal thoughts or daydreaming—distant memories and concepts that would not normally connect become more likely to link [23]. This demonstrated the importance of mind-wandering during the idea-generation stage. Mind-wandering is conceptualized as a thinking style that contributes to creativity.

In addition, the dual pathway to creativity model has emerged as an alternative perspective on thinking styles in creativity has emerged [24]. This model conceptualizes creativity as having two pathways: cognitive flexibility [25], which involves simultaneously processing diverse concepts across categories, and cognitive persistence, which involves sustained, deep processing of a small number of concepts. These theoretical frameworks as well as methodological considerations guided the selection of personality, thinking styles, and motivation as the primary domains for C-PETS. First, these three components are inherently individual-difference variables that can be reliably assessed through self-report psychological scales [12, 26]. Second, and importantly for applied purposes, these constructs are considered malleable through education and training [27, 28], suggesting that the scale could serve as a foundation for creativity-enhancing interventions.

### Rationale for the selection of criterion measures

This study developed the C-PETS, capable of measuring creativity multifacetedly. To achieve this objective, new items for measuring creativity were developed by referencing questionnaire items shown to be associated with creativity in previous research. The scale was developed through exploratory factor analysis (EFA). To establish criterion-related validity, the association with creative self-efficacy was first investigated. The Short Scale of Creative Self-Efficacy (SSCS) [29–31] is a self-assessment of creative ability and potential. Creative self-efficacy refers to the belief or confidence in one’s ability to produce creative outcomes. The SSCS consists of two sub-factors: Creative Self-Efficacy (CSE) and Creative Personal Identity (CPI). CSE is the overall belief in one’s ability to engage in creative thinking and problem-solving. CPI refers to the extent to which creativity is treated as an important part of one’s personal identity [31, 32]. The SSCS has been shown to correlate with self-reported creativity, indicating how creative an individual perceives themselves to be, and with the Work Preference Inventory [33], which measures motivation in the workplace. This study anticipates a positive correlation between the developed questionnaire and the SSCS.

Subsequently, the association between the C-PETS and the Aesthetic Experiences Scale (AES) [34] was investigated. The AES assesses the depth of physiological and emotional responses elicited by exposure to artworks or music. AES scores, which measure the frequency of aesthetic experiences, are closely associated with creative achievement. Silvia and Nusbaum [34] demonstrated that the personality underlying the AES strongly predicts artistic creativity. Therefore, a positive correlation is expected between the developed questionnaire and the AES.

Finally, the association between the C-PETS and Divergent Association Task (DAT)—a measure of divergent thinking and a key component of creativity—was investigated. In the DAT, participants are asked to list 10 words that are as different from each other as possible. The DAT’s scoring algorithm estimates and scores the average semantic distance between words. Higher scores are assigned for greater semantic distance, and the test is considered to measure divergent thinking and verbal creativity [35]. Previous research has shown a weak positive correlation (*r* = .23) between the DAT and CSE [36]. This result suggests that the divergent thinking measured by the DAT may constitute only a small part of the creative process [36]. However, examining the correlation with the DAT enables assessment of the correlation between the developed measure and divergent thinking. Based on prior findings, a weak positive correlation between the DAT and the developed measure is anticipated.

### Examination of creativity-related individual differences

Differences in creativity scores based on age, gender, and educational attainment were examined to illustrate the practical utility of the scale in exploring group differences. Palmiero et al. [37] compared association fluency, production fluency, flexibility, and originality between a young group (17–22 years) and a middle-aged group (40–50 years). No significant difference was found in association fluency, but middle-aged individuals performed comparably to the young group in production fluency, flexibility, and originality. Their findings [37] indicate that young adulthood (20–35 years) is associated with a peak in divergent thinking, which then stabilizes during middle age (40–50 years). Therefore, the current study examines age differences in scores on the C-PETS to verify whether there are significant age-related differences in creativity. Based on these prior findings, creativity scores were expected to be maintained through middle age.

Second, regarding gender differences in creativity, recent empirical evidence and a large-scale meta-analysis indicate that overall creative potential is relatively equivalent between men and women [38, 39]. While global scores show minimal gender differences across diverse contexts [39], subtle variations can emerge when examining specific creative styles or sub-factors rather than overarching capacity [38, 39].

Furthermore, the relationship between educational attainment and creativity scores was investigated. While traditional creativity research has focused on divergent thinking, Cropley [28] argued that convergent thinking is an essential component of creativity, particularly in the evaluation and refinement of ideas. He [28] posited that convergent thinking is often fostered through formal education. Given that convergent thinking is crucial in the convergence to verification stage of the creative process [40], the significance of formal education was emphasized.

Finally, adults with university-level education or higher tend to show higher motivation to learn [41]. Thus, a positive correlation with educational attainment was anticipated, as the specialized knowledge and evaluative judgment skills (aspects of convergent thinking) cultivated through higher education form the foundation for fostering creative potential. This study therefore examines differences in creativity scores based on the highest level of educational attainment.

## Research questions

To achieve the objective of developing and validating a multifaceted measure of creativity, this study addresses the following research questions (RQs):

- RQ1: What is the factor structure of the C-PETS, and does it demonstrate sufficient construct validity?
- RQ2: To what extent does the C-PETS correlate with existing relevant scales, and does it possess adequate criterion-related validity?
- RQ3: Does the C-PETS possess adequate psychometric reliability, including internal consistency and test–retest reliability?
- RQ4: Does the developed scale demonstrate practical utility by capturing differences based on demographic attributes, such as age, gender, and educational attainment?

## Study 1

### Method

#### Objectives

The primary objective of this study was to develop the C-PETS by referencing established scale items that measure personality, thinking styles, and motivation, which are theoretically associated with creativity.

#### Participants

The initial sample size was 600. A total of 28 responses with ceiling and floor effects were excluded from the analysis. The final sample for EFA comprised 572 participants (age: *M* = 39.94, *SD* = 11.48). Participants self-identified their ethnicities as White (67%), Black or African American (16%), Hispanic (12%), Asian (5%), or other (less than 1%). Data were collected in July 2025.

The final sample for confirmatory factor analysis (CFA), recruited separately from the EFA sample, comprised 943 participants (age: *M* = 38.76, *SD* = 11.44). Participants self-identified their ethnicities as White (62%), Black or African American (19%), Hispanic (11%), Asian (4%), or other (less than 4%). Data were collected in August 2025.

#### Procedures

An online survey was conducted among participants registered with an online survey company (Cross Marketing, Inc.) in North America. Respondents were informed that ethical approval was obtained from the appropriate research ethics committee and that completing the survey constituted their consent to participate in the study.

A stratified sampling approach was employed using eight demographic blocks based on four age categories (18–29, 30–39, 40–49, and 50–59 years) and two gender categories (men and women). This recruitment method was consistently applied across all sub-studies. The participants for each study were recruited separately.

Following Comrey and Lee’s [42] recommendations for sample size in scale development studies (100 = poor, 200 = fair, 300 = good, 500 = very good, and ≥ 1,000 = excellent), the target sample size was set to 500 or more, and participants were recruited by ensuring no bias in either gender or age.

#### Scale development

Once EFA was conducted, a separate sample was recruited for CFA. The initial sample size was 1,000. Fifty-seven responses that showed ceiling/floor effects and identical response patterns across all items were excluded from the analysis. Additional analyses were conducted to verify the higher-order models.

The initial C-PETS item pool (167 items) was compiled by selecting items from established psychological scales empirically linked to creativity (Additional File 1). These source measures included items for openness to experience [43], adaptive perfectionism [44], cognitive flexibility [25], tolerance for ambiguity [14], divergent thinking and related daily actions [15, 45], the regulation of cognition factor of metacognitive awareness [46], absorption, and specific and diffuse curiosity [47], need for cognition [22], and mind-wandering [48]. Cognitive persistence within the dual pathway to creativity model was assessed using items related to the need for cognition and curiosity [22, 47].

The qualitative screening process to reduce the initial item pool from 167 to the 42-item preliminary version was conducted exclusively by the author, who is a licensed psychologist with a Ph.D. and possesses extensive expertise in psychometric development and the validation of cognitive process scales. To ensure theoretical consistency and minimize potential subjective bias, the author applied a systematic internal auditing process based on predefined conceptual criteria. Each item was rigorously evaluated against the operational definitions of the three targeted domains—personality, thinking styles, and motivation—and retained only if it clearly and uniquely mapped onto a single construct. Items that simultaneously reflected multiple constructs or exhibited conceptual ambiguity across categories were strictly excluded to maintain the structural purity and unidimensionality of the scale. Furthermore, a content redundancy check was performed to remove items with nearly identical semantic meaning across different source scales, ensuring a parsimonious final pool. Although inter-rater agreement was not tested owing to the single-author nature of this study, this expert-driven screening ensured that only the most theoretically sound and linguistically clear items proceeded to the empirical validation phase in Study 1.

This study focused specifically on domain-general aspects that influence creative potential across contexts. Items were also excluded if they did not align sufficiently with the specific operational definitions of the three domain-general components.

After removing items with nearly identical content, a preliminary 42-item version was created. Participants responded to the items using a 5-point Likert scale ranging from 1 (“strongly disagree”) to 5 (“strongly agree”).

#### Statistical analysis

All statistical analyses were conducted using IBM SPSS version 25 and Amos 26.

## Results

EFA was conducted using maximum likelihood extraction with Promax rotation on the initial 42-item pool (Table 1). Items with similar content, high inter-item correlations, and those that were difficult to interpret were excluded from the analysis.

**Table 1:** Initial C-PETS item pool and sources

No.	Item	Category	Construct	Reference
1	I have many rough ideas.	Divergent thinking	Thinking styles	[15, 45]
2	I am good at combining ideas in ways that others have not tried before.	Divergent thinking	Thinking styles	[15, 45]
3	I come up with new ideas by building on others’ ideas.	Divergent thinking	Thinking styles	[15, 45]
4	When I come up with a new idea, I get really excited.	Divergent thinking	Thinking styles	[15, 45]
5	I reflect on important information as I come across it.	Metacognitive Awareness	Thinking styles	[46]
6	I consciously direct my attention toward important information.	Metacognitive Awareness	Thinking styles	[46]
7	I provide my own examples to make information more meaningful.	Metacognitive Awareness	Thinking styles	[46]
8	I draw diagrams to enhance my understanding.	Metacognitive awareness	Thinking styles	[46]
9	I connect new information to what I already know.	Metacognitive awareness	Thinking styles	[46]
10	I focus on the overall meaning rather than on specific details.	Metacognitive awareness	Thinking styles	[46]
11	I research unclear information until I fully understand it.	Need for cognition	Motivation	[22]
12	I prefer complex problems to simple ones.	Need for cognition	Motivation	[22]
13	I like to take on new challenges.	Curiosity	Motivation	[47]
14	I am very interested in doing things that no one has done before.	Curiosity	Motivation	[47]
15	I have an interest in many things.	Curiosity	Motivation	[47]
16	I keep thinking until I reach a clear and precise answer.	Curiosity	Motivation	[47]
17	When I learn new things, I want to research them thoroughly.	Curiosity	Motivation	[47]
18	When an unexpected event occurs, I investigate it until I determine the cause.	Curiosity	Motivation	[47]
19	I let my thoughts wander on purpose.	Mind-wandering	Thinking styles	[48]
20	I let my mind wander even when I am supposed to be doing something else.	Mind-wandering	Thinking styles	[48]
21	When my mind wanders, it tends to jump from one to another.	Mind-wandering	Thinking styles	[48]
22	I decline invitations that are not beneficial for me.	Adaptive perfectionism	Personality	[44]
23	People say I am hard on myself.	Adaptive perfectionism	Personality	[44]
24	I hold the highest standards.	Adaptive perfectionism	Personality	[44]
25	It is important to set high standards for myself.	Adaptive perfectionism	Personality	[44]
26	I tend to become absorbed in one thing.	Absorption	Personality	[47]
27	When I become interested in something, I become completely absorbed in it.	Absorption	Personality	[47]
28	My imagination is sparked by the unfamiliar.	Ambiguity tolerance	Personality	[14]
29	It is interesting to interpret things in many different ways.	Ambiguity tolerance	Personality	[14]
30	Imperfections are interesting.	Ambiguity tolerance	Personality	[14]
31	To some extent, I am able to accept imperfections.	Ambiguity tolerance	Personality	[14]
32	I trust my creativity.	Divergent thinking	Thinking styles	[15, 45]
33	When thinking about things, I try to understand the bigger picture.	Cognitive flexibility	Thinking styles	[25]
34	I am willing to listen and consider alternatives for solving problems.	Cognitive flexibility	Thinking styles	[25]
35	I would prefer to consider several ways to tackle a problem.	Cognitive flexibility	Thinking styles	[25]
36	I have a diverse range of ideas.	Cognitive flexibility	Thinking styles	[25]
37	I review my work for mistakes.	Cognitive flexibility	Thinking styles	[25]
38	I can work on many things at once.	Cognitive flexibility	Thinking styles	[25]
39	I can shift my focus between two different tasks.	Cognitive flexibility	Thinking styles	[25]
40	I can quickly direct my focus to a single task when needed.	Cognitive flexibility	Thinking styles	[25]
41	I value inspiration.	Openness to experience	Personality	[43]
42	I can convert an object with a specific purpose into one with a different function.	Divergent thinking	Thinking styles	[15, 45]

Note. *C-PETS* Creative Personality and Thinking Styles Scale

**Fig. 1:**
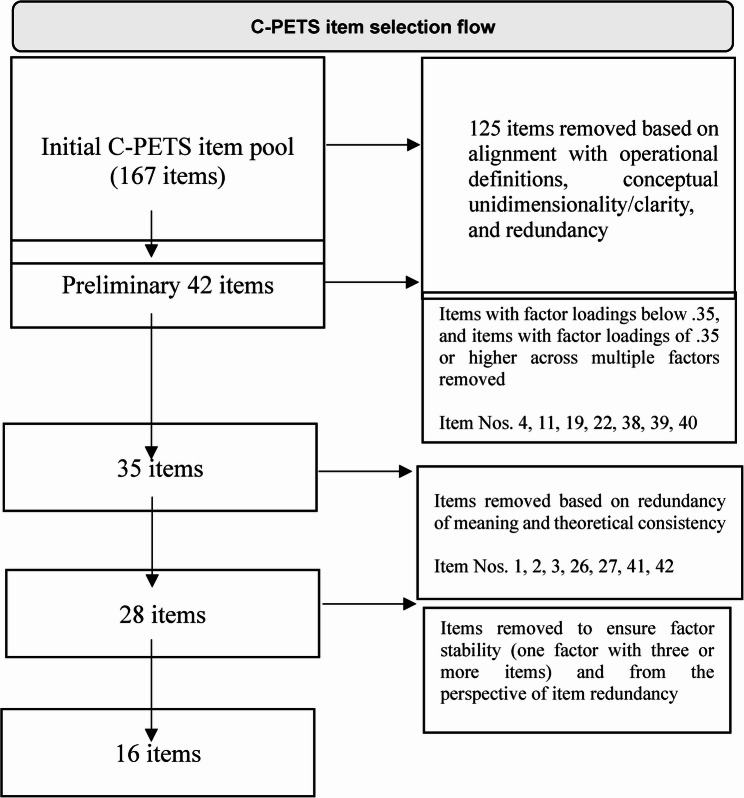
Flowchart of C-PETS development

The scree plot, eigenvalues, parallel analysis, and minimum average partial correlation indicated a five-factor solution. The items were retained based on the following criteria: (1) factor loadings of 0.35 or higher [49], (2) interpretability within the factor structure, (3) absence of cross-loadings exceeding 0.35 on multiple factors [49], and (4) a concise number of items without redundancy, considering respondent burden. Items that failed to meet these criteria were excluded (Fig. 1).

The final EFA resulted in a five-factor, 16-item version of the C-PETS (Table 2). The five factors were labeled based on their item content as follows: Broad Thinking (BT), comprising items representing flexibility and broad thinking styles; Thorough Thinking (TT), consisting of items representing thorough and systematic thinking styles; Information Manipulation (IM), including items representing the ability to connect information to deepen knowledge and manipulate acquired information for better understanding; Challenge-Seeking (CS), comprising items representing thoughts and motivations related to seeking challenges; and Ambiguity Tolerance (AT), consisting of items representing attitudes that accept incomplete states and information.

**Table 2:** Exploratory factor analysis results: factor loadings and commonalities for C-PETS items

Item	EFA factors					Communality
	1	2	3	4	5	
Broad Thinking						
When thinking about things, I try to understand the bigger picture.	**.824**	−.061	–.058	.028	.050	.632
I would prefer to consider several ways to tackle a problem.	**.790**	–.001	.039	–.104	–.009	.574
I have a diverse range of ideas.	**.638**	–.020	–.009	.149	.030	.532
I am willing to listen and consider alternatives for solving problems.	**.608**	.051	.030	–.059	.103	.503
Thorough Thinking						
When an unexpected event occurs, I investigate it until I determine the cause.	–.062	**.768**	–.028	.035	.052	.577
When I learn new things, I want to research them thoroughly.	–.042	**.668**	.099	.066	.019	.592
I keep thinking until I reach a clear and precise answer.	.171	**.517**	.108	.125	–.159	.546
Information Manipulation						
I connect new information to what I already know.	–.001	–.077	**.809**	.041	.000	.605
I provide my own examples to make information more meaningful.	–.034	–.026	**.683**	.051	.060	.550
I reflect on important information as I come across it.	.059	.193	**.560**	–.136	.073	.512
Challenge-Seeking						
I like to take on new challenges.	.045	–.036	.043	**.774**	.019	.655
I prefer complex problems to simple ones.	–.128	.021	–.082	**.724**	.137	.484
I am very interested in doing things that no one has done before.	.098	.155	.106	**.525**	–.133	.517
Ambiguity Tolerance						
Imperfections are interesting.	.045	–.061	.065	.136	**.657**	.580
It is interesting to interpret things in many different ways.	.162	.320	–.123	–.011	**.478**	.572
To some extent, I am able to accept imperfections.	.209	.087	.123	–.050	**.473**	.400

The numbers in bold indicate the meaning of the numbers that make up the factor

Note. *C-PETS* Creative Personality and Thinking Styles Scale

After EFA, CFA was performed on the separate sample of 943 participants to assess model fit. CFA of the five-factor model demonstrated excellent fit indices: GFI = .95, CFI = .96, RMSEA = .05, AIC = 421.57. These results indicated that the five-factor structure provided a good fit to the data. Internal consistency reliability coefficients (Cronbach’s α) for each factor and the total scale were as follows: BT (α = .76), TT (α = .73), IM (α = .77), CS (α = .71), AT (α = .74), and C-PETS (α = .91). All reliability coefficients exceeded the acceptable threshold of 0.70, indicating adequate internal consistency. Furthermore, item-level descriptive statistics and an inter-item correlation matrix were examined to ensure measurement stability and the absence of item redundancy (Additional File 2). All items demonstrated appropriate mean scores and variances, and inter-item correlations remained within an acceptable range (*r* < .80), confirming that each item contributed uniquely to its respective factor without excessive overlap.

**Fig. 2:**
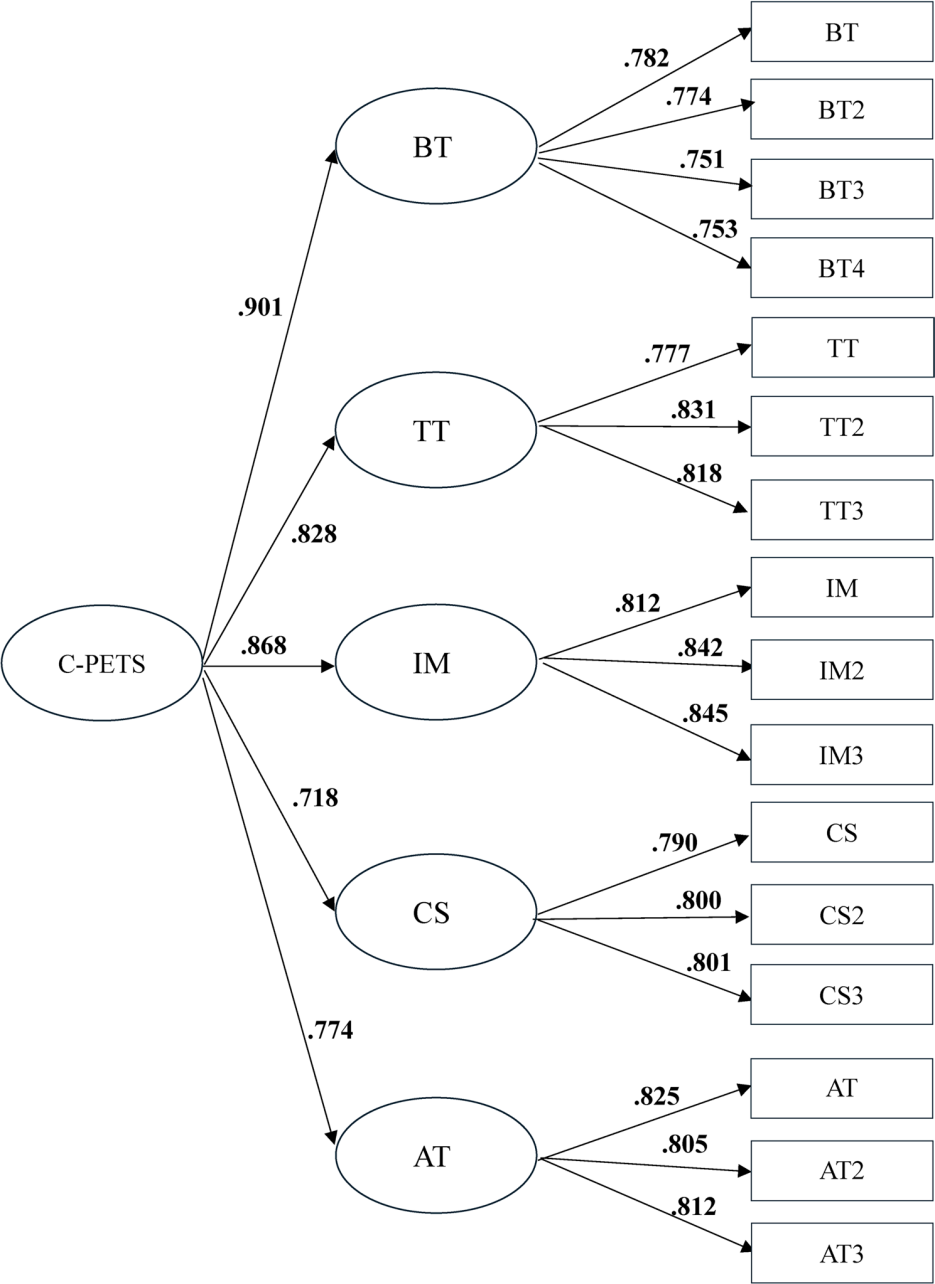
Higher-order path diagram. Note. AT, Ambiguity Tolerance; BT, Broad Thinking; C-PETS, Creative Personality and Thinking Styles Scale; CS, Challenge-Seeking; IM, Information Manipulation; TT, Thorough Thinking

After CFA, an analysis was performed to verify the higher-order model. The results were as follows: GFI = .97, CFI = .98, RMSEA = .02, AIC = 291.8. The model fit was good for the values obtained from both CFA and the higher-order model. Because the goal of this study was to use the C-PETS as a multifaceted measure encompassing personality, thinking style, and motivation, rather than using the factors constituting each scale individually, the higher-order model was adopted (Fig. 2).

Correlation analyses revealed moderate to strong relationships between the factors and the total C-PETS score. The correlation coefficients were as follows: BT–TT (*r* = .71), BT–IM (*r* = .77), BT–CS (*r* = .53), BT–AT (*r* = .63), TT–IM (*r* = .68), TT–CS (*r* = .46), TT–AT (*r* = .55), IM–CS (*r* = .52), IM–AT (*r* = .58), and CS–AT (*r* = .39). The correlations between each factor and the total C-PETS score were significant: BT–C-PETS (*r* = .90), TT–C-PETS (*r* = .82), IM–C-PETS (*r* = .86), CS–C-PETS (*r* = .71), and AT–C-PETS (*r* = .77). These findings indicate that all factors contributed substantially to the overall construct while maintaining reasonable independence from one another.

## Study 2

### Objectives

The primary objective of Study 2 was to examine the criterion-related validity of the C-PETS developed in Study 1 by conducting correlation analyses between the C-PETS, SSCS, AES, and DAT.

### Method

#### Participants

The initial sample size was 200. Participants were recruited by ensuring no bias in either gender or age. Following the same recruitment procedure as in Study 1, 37 responses showing ceiling and floor effects were found in all three questionnaires. DAT responses that contained data consisting of sentences rather than individual words, or meaningless elements such as numbers and letters, were excluded from the analysis. In total, 163 participants completed the survey (age: *M* = 39.74, *SD* = 11.17). Participants self-identified their ethnicities as White (68%), Black or African American (18%), Hispanic (9%), Asian (3%), or other (2%). Data were collected in August 2025.

#### Criterion-related validity assessment

Correlations with three theoretically related measures were investigated to examine the criterion-related validity of the C-PETS. The internal consistency reliabilities of the subscales and total instrument were BT (α = .71), TT (α = .69), IM (α = .70), CS (α = .66), AT (α = .69), and C-PETS (α = .90).

#### Creative self-efficacy

The SSCS [29–31] was used to assess creative self-efficacy, which refers to beliefs about one’s ability to produce creative outcomes and includes the self-evaluation of creative abilities and potential. The SSCS comprises two subscales: CSE, which represents overall beliefs about one’s abilities in creative thinking and problem-solving, and CPI, which indicates the extent to which creativity is treated as an important part of personal identity [32]. Previous research has demonstrated correlations between the SSCS and self-reported creativity measures as well as workplace motivation measured using the Work Preference Inventory [33]. Internal consistency reliability coefficients for the SSCS were (α = .92), CPI (α = .90), and CSE (α = .86).

#### Aesthetic experiences

The AES [34] was used to measure aesthetic experiences. This scale assesses the frequency of aesthetic chills, described as “a feeling of goosebumps and shivers down the spine” when encountering artistic works [34, 50]. Individuals who frequently experience aesthetic chills have been shown to score higher on openness to experience in the Big Five personality model [51]. Kenett et al. [52] suggested that creativity and aesthetic experiences are related to openness to experience. The internal consistency reliability was α = 0.86.

#### Divergent thinking

The DAT [35] was used to assess divergent thinking, a component of creativity. In the DAT, participants were asked to generate 10 words that were as different from each other as possible. The computational algorithm of the task estimates the average semantic distance between words for scoring, with greater semantic distances receiving higher scores. The DAT measures divergent thinking and verbal creativity. Previous research has shown a weak positive correlation (*r* = .23) between the DAT and CSE [36], suggesting that divergent thinking, as measured using the DAT, may constitute only a small portion of the creative process. However, examining the correlations with the DAT allows for the investigation of the relationship between the C-PETS and divergent thinking.

### Results

#### Criterion-related validity

Correlation analyses were conducted to examine the relationship between the C-PETS and validation measures (Table 3). Strong positive correlations were found between the C-PETS and the SSCS measures: C-PETS and CSE (*r* = .76, *p* < .01), C-PETS and CPI (*r* = .66, *p* < .01), and C-PETS and total SSCS scores (*r* = .75, *p* < .01). A moderate positive correlation was observed between the C-PETS and AES (*r* = .30, *p* < .01). A weak but significant correlation was observed between the C-PETS and DAT (*r* = .18, *p* < .05) (Table 3).

**Table 3:** Means, standard deviations, and correlations between the C-PETS, SSCS, AES, and DAT

Item	*M*	*SD*	1	2	3	4	5	6	7	8	9	10	11
1. C-PETS	64.03	9.94	ー	.91**	.83**	.85**	.73**	.75**	.76**	.66**	.75**	.30**	.18*
2. BT	16.87	2.66		ー	.77**	.81**	.55**	.63**	.74**	.63**	.72**	.29**	.21**
3. TT	12.20	2.25			ー	.68**	.54**	.46**	.66**	.52**	.63**	.21**	.12
4. IM	12.40	2.12				ー	.44**	.62**	.59**	.50**	.58**	.27**	.16*
5. CS	10.58	2.73					ー	.40**	.59**	.54**	.60**	.17*	.06
6. AT	11.95	2.32						ー	.53**	.49**	.54**	.29**	.20**
7. CSE	23.74	4.90							ー	.79**	.94**	.24**	.11
8. CPI	19.59	4.93								ー	.94**	.23**	.11
9. SSCS	43.34	9.31									ー	.25**	.12
10. AES	40.21	11.15										ー	.06
11. DAT	71.49	10.59											ー

Note. ** *p* < .01, * *p* < .05; *M* mean, *SD* standard deviation, *AT* Ambiguity Tolerance, *BT* Broad Thinking, *C-PETS* Creative Personality and Thinking Styles Scale, *CS* Challenge-Seeking, *SSCS* Short Scale of Creative Self-Efficacy, *CPI* Creative Personal Identity, *DAT* Divergent Association Task, *IM* Information Manipulation, *TT* Thorough Thinking

## Study 3

### Objectives

The test–retest reliability of the C-PETS was examined by re-administering the scale to the same participants after two weeks to assess the measure’s temporal stability.

### Method

#### Participants

The initial sample size was 101. Participants were recruited by ensuring no bias in either gender or age. Only the responses of participants who answered the survey again two weeks later were considered. No ceiling or floor effects and identical responses were observed. In total, 101 participants completed the survey (age: *M* = 39.93, *SD* = 11.05). Participants self-identified as White (58%), Black or African American (27%), Hispanic (9%), Asian (4%), or other (2%). Data were collected in August 2025. Participants in Study 2 were different from those in Study 3.

#### Test–retest reliability

The test–retest reliability analysis with a two-week interval demonstrated strong temporal stability of the C-PETS (*r* = .87), indicating that the scale produced consistent results over time.

## Study 4

### Objectives

The objective of Study 4 was to examine whether there were differences in C-PETS scores based on educational background and age differences in scale performance.

### Method

#### Participants

The initial sample size was 1,000. Participants were recruited by ensuring no bias in either gender or age. Following the same recruitment procedure as in Study 1, 46 responses displaying ceiling and floor effects were excluded. In total, 954 participants completed the survey (age: *M* = 38.88, *SD* = 11.54). Participants self-identified their ethnicities as White (61%), Black or African American (21%), Hispanic (10%), Asian (6%), or other (2%). Data were collected in August 2025.

#### Reliability analysis

Internal consistency reliability coefficients for the C-PETS subscales and total scale were as follows: BT (α = .78), TT (α = .74), IM (α = .75), CS (α = .69), AT (α = .74), and C-PETS (α = .91).

#### Statistical analysis

To examine the relationship between educational attainment and C-PETS scores, educational attainment was treated as an ordinal variable and coded into three levels: 1 = high school, 2 = university, and 3 = graduate school. Consequently, Spearman’s rank correlation coefficients (*ρ*) were calculated to assess the associations between educational attainment and each subscale, as well as the total C-PETS score. A one-way ANOVA was performed to examine differences in the mean values of each indicator (BT, TT, IM, CS, AT, Total) across the four age groups (1–4). For variables showing a significant main effect, post-hoc comparisons using Tukey’s HSD method were conducted to examine specific intergroup differences. For men and women, the Shapiro–Wilk test was performed, and normality was rejected for all variables (*p* < .05). Levene’s test for equality of variances was also conducted. Considering these results, the Mann–Whitney U test, a nonparametric test, was used to compare the differences in mean values between men and women.

### Results

#### Educational attainment differences

**Fig. 3:**
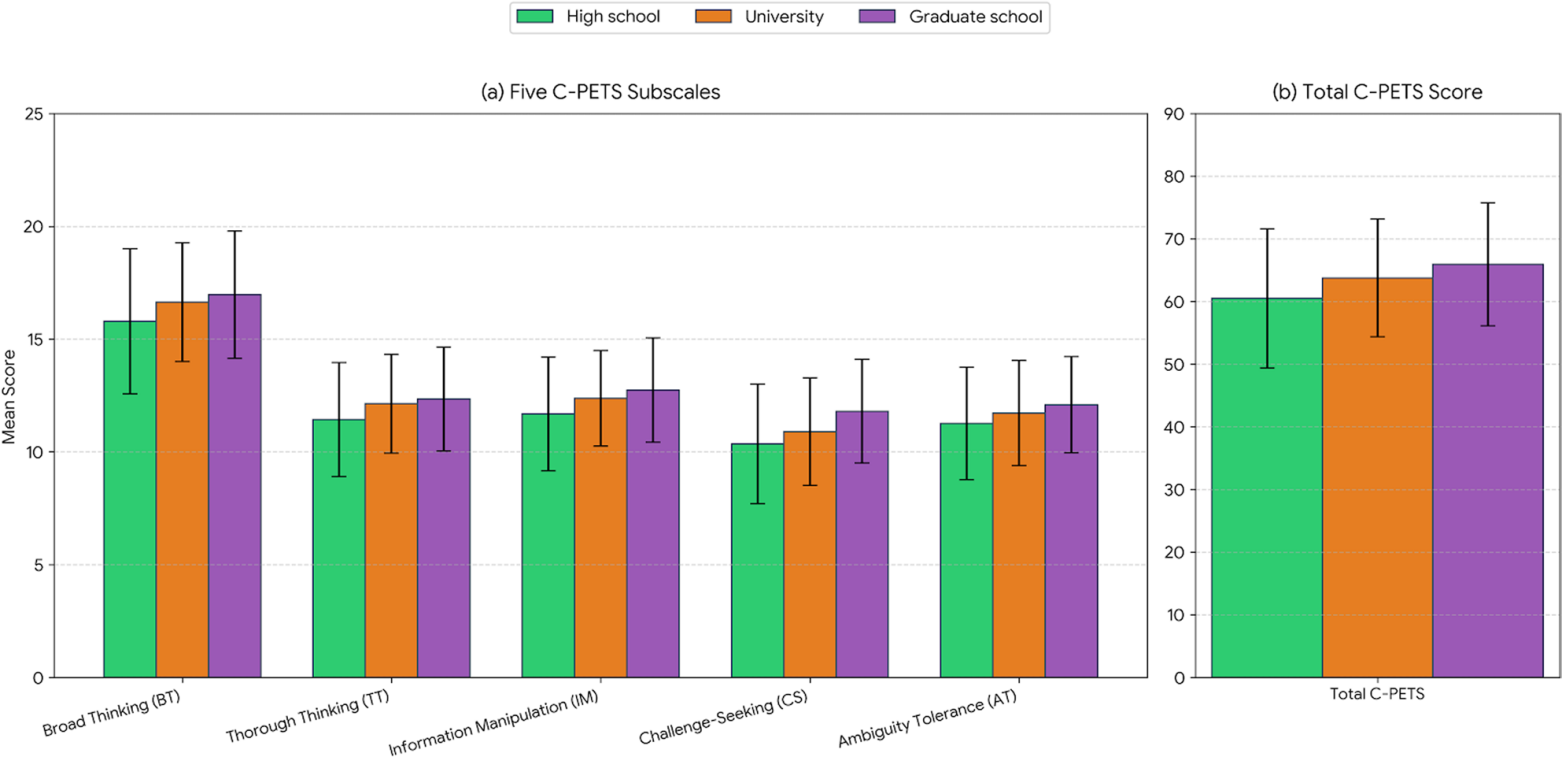
Differences in C-PETS scores by educational attainment. **a** Comparison of mean scores for the five C-PETS subscales: Broad Thinking (BT), Thorough Thinking (TT), Information Manipulation (IM), Challenge-Seeking (CS), and Ambiguity Tolerance (AT). **b** Comparison of mean scores for the total C-PETS scale. Error bars represent standard deviations. All variables show a consistent upward trend as educational attainment level increases

As shown in Table 4; Fig. 3, all variables exhibited statistically significant positive correlations with educational attainment (all *ps* < 0.001 after correction). Specifically, Fig. 3a highlights the consistent increase across all five sub-factors, while Fig. 3b illustrates the overall enhancement of creative potential associated with higher levels of formal education.

**Table 4:** Means, standard deviations, and Spearman’s rank correlations of C-PETS scores across educational attainment

Item	High school (*n* = 464)		University (*n* = 339)		Graduate school (*n* = 151)		Spearman’s *ρ*	Corrected *p*-value
	*M*	SD	*M*	*SD*	*M*	*SD*		
BT	15.79	3.21	16.64	2.63	16.97	2.82	.16	< 0.001
TT	11.43	2.52	12.13	2.19	12.34	2.31	.16	< 0.001
IM	11.68	2.52	12.37	2.12	12.74	2.31	.19	< 0.001
CS	10.35	2.65	10.9	2.38	11.80	2.30	.19	< 0.001
AT	11.26	2.50	11.72	2.33	12.09	2.13	.13	< 0.001
C-PETS	60.50	11.09	63.76	9.39	65.94	9.83	.21	< 0.001

Note. *M* mean, *SD* standard deviation, *AT* Ambiguity Tolerance, *BT* Broad Thinking, *C-PETS* Creative Personality and Thinking Styles Scale, *CS* Challenge-Seeking, *IM* Information Manipulation, *TT* Thorough Thinking

#### Age and gender differences

To examine score differences across the four age groups (18–29, 30–39, 40–49, 50–59) on each measure, a one-way ANOVA followed by Tukey’s multiple comparisons test was conducted. The analysis revealed a significant main effect for BT (*F*(3, 950) = 4.62, *p* < .01, *η*^2^ = .01). Post hoc comparisons revealed that the mean score for the 50–59 age group was significantly higher than the mean scores for the 18–29 (*p* < .01) and 30–39 (*p* < .05) age groups. A significant main effect was also found for TT (*F*(3, 950) = 3.01, *p* < .05, *η*^2^ = .01). Multiple comparisons revealed that the scores of the 50–59 age group were significantly higher than those of the 18–29 age group (*p* < .05).

However, no significant main effects were observed for the following scales: IM (*F*(3, 950) = 2.02, *p* = .10), CS (*F*(3, 950) = 1.54, *p* = .20), AT (*F*(3, 950) = 1.18, *p* = .31), and total (*F*(3, 950) = 1.55, *p* = .20). These results suggest that no significant differences by age group exist for scales other than BT and TT (Table 5).

**Table 5:** Means and standard deviations for C-PETS subscales and total score across age groups

Item	Age 18–29 (*n* = 244)		Age 30–39 (*n* = 234)		Age 40–49 (*n* = 250)		Age 50–59 (*n* = 226)		*F *(3,950)	*p*-value	Effect size
	*M*	*SD*	*M*	*SD*	*M*	*SD*	*M*	*SD*			
BT	15.97^a^	3.34	16.03^a^	3.21	16.24	2.86	16.89^b^	2.35	4.62	0.01	0.01
TT	11.48^a^	2.60	11.77	2.46	11.94	2.30	12.11^b^	2.09	3.01	0.03	0.01
IM	11.91	2.61	12.03	2.40	12.03	2.20	12.40	1.84	2.02	0.10	0.01
CS	10.84	2.64	10.79	2.50	10.94	2.44	10.46	2.60	1.54	0.20	0.00
AT	11.42	2.55	11.39	2.53	11.73	2.30	11.65	2.19	1.18	0.31	0.00
C-PETS	61.64	11.72	62.03	11.14	62.90	10.24	63.54	8.54	1.55	0.20	0.00

Note. Values are presented as mean and standard deviation. *BT* Broad Thinking, *TT* Thorough Thinking, *IM* Information Manipulation, *CS* Challenge-Seeking, *AT* Ambiguity Tolerance. Means with different superscripts (a, b) in the same row indicate significant differences at the *p* < .05 level based on Tukey’s HSD post hoc test

The analysis revealed significant gender differences in both CS and AT. Men (*M* = 11.04, *SD* = 2.60) scored significantly higher in CS than women (*M* = 10.52, *SD* = 2.49; *U* = 128838.0, *p* < .001). In contrast, women scored significantly higher than men in AT (*U* = 101678.0, *p* = .004). However, no significant gender differences were observed in BT (*U* = 110040.0, *p* = .38), TT (*U* = 115022.5, *p* = .75), IM (*U* = 109072.0, *p* = .27), or C-PETS (*U* = 114418.5, *p* = .86) (Table 6).

**Table 6:** Comparison of C-PETS scores by gender: means, standard deviations, and Mann–Whitney U test results

Item	Men (*n* = 466)		Women (*n* = 488)		U-value	*p*-value
	*M*	*SD*	*M*	*SD*		
BT	16.15	3.09	16.40	2.90	110040.0	.385
TT	11.82	2.45	11.83	2.33	115022.5	.754
IM	11.98	2.38	12.20	2.21	109072.0	.271
CS	11.04	2.60	10.52	2.49	128838.0	.001
AT	11.32	2.47	11.78	2.33	101678.0	.004
C-PETS	62.30	11.04	62.73	10.00	114418.5	.867

Note. *M* mean, *SD* standard deviation, *AT* Ambiguity Tolerance, *BT* Broad Thinking, *C-PETS* Creative Personality and Thinking Styles Scale, *CS* Challenge-Seeking, *IM* Information Manipulation, *TT* Thorough Thinking

## Discussion

This study successfully developed the C-PETS, which measures personality, thinking styles, and motivation—constructs that are theoretically associated with creativity. Through EFA, a five-factor, 16-item scale was developed that demonstrated good model fit and adequate test–retest reliability.

### Factor structure and theoretical implications

The factor structure of the C-PETS provides insights into the relationships among creativity-related cognitive processes. Traditionally, divergent and convergent thinking have been viewed as opposing cognitive processes, leading to debates on why seemingly contradictory approaches are necessary for creativity. However, Eysenck [53] argued that both convergent and divergent thinking require associations with multiple ideas, suggesting that these cognitive processes are not fundamentally different. Furthermore, Eymann et al. [54] proposed that divergent and convergent thinking exist on a continuum rather than as a dichotomy. In this study, these processes were represented by BT, which reflects flexibility and broad thinking styles, and TT, which represents systematic thinking approaches. The positive correlation between BT and TT indicates that although they are distinct cognitive styles, they share common variance as integral components of an individual’s creative resources. Rather than providing definitive evidence for a continuity of cognitive processes, these results, coupled with the supported higher-order model, suggest that these factors function as interrelated yet independent facets that collectively contribute to a multifaceted creative personality and thinking style.

Similarly, tolerance for ambiguity and convergent thinking, which might appear contradictory, were represented by AT and TT, respectively. The positive correlation between these factors can be explained by the stage-dependent nature of the creative processes. Tolerance for ambiguity is essential during the early stages of the creative process when divergent thinking generates multiple ideas, whereas convergent thinking becomes necessary during the evaluation and selection phases of generated ideas [54]. This stage-specific requirement explains why these seemingly opposing factors exhibited positive correlations in the C-PETS structure.

### Reliability and temporal stability of the C-PETS

Regarding RQ2, the C-PETS demonstrated robust internal consistency and temporal stability across multiple studies. Cronbach’s α coefficients for the total scale consistently reached 0.90 or higher in Study 1, Study 2, and Study 4. While the alpha coefficients for individual subscales varied slightly, most values were above or near the acceptable threshold of 0.70, indicating that the five factors possess adequate internal consistency. Furthermore, the two-week test–retest reliability coefficient was strong (*r* = .87), providing evidence for the measure’s temporal stability. These results suggest that the C-PETS is a psychometrically sound scale capable of providing stable and consistent assessments of creativity-related personality and thinking styles over time.

### Construct and criterion-related validity and relationships with existing measures

The criterion-related validity of the C-PETS was supported by its positive correlations with the SSCS, AES, and DAT. The strong correlation with the SSCS aligns with previous research demonstrating a relationship between CSE and creativity-related constructs. Vujović and Šupić [55] showed that CSE positively correlates with the tendency to seek new experiences, which corresponds to CS in the C-PETS, representing motivation for seeking challenges and new experiences.

The relationship between the C-PETS and AES provides additional evidence of validity. The AES focuses on unique experiences in aesthetic contexts and has been linked to openness to experience in the Big Five personality model. Research suggests that aesthetic evaluation involves a general system centered on the mesolimbic reward circuit for evaluating the hedonic value of all sensory objects [56], with dopamine playing a crucial role. Importantly, dopamine can be activated by information, independent of external rewards [57]. This reward-independent dopamine function relates to openness to experience, which has been characterized as “motivated cognitive flexibility” [58]. Because the C-PETS was developed with reference to openness to experience and cognitive flexibility, its positive correlation with the AES, which is associated with openness to experience, provides theoretical support for the scale’s validity.

A significant positive correlation was observed between the C-PETS and DAT. This finding is consistent with previous research reporting significant correlations between divergent thinking tests and certain self-report measures of creativity, such as the SSCS [36]. While some studies suggest a discrepancy between self-report questionnaires and performance-based tests—attributing it to the difference between measuring self-perception versus actual cognitive performance—the current results indicate that the C-PETS successfully captures the divergent thinking processes assessed by such tests. Therefore, the correlation between the C-PETS and the DAT in this study provides further evidence for the concurrent validity of the C-PETS as a measure of creative potential.

The relationship between educational attainment and C-PETS scores provided evidence of theoretical support for the scale’s application. Existing literature demonstrates that a longer educational history correlates with greater openness to experience [56]. Because the development of the C-PETS incorporated items related to openness to experience, and openness to experience has been correlated with cognitive flexibility [25], curiosity types [59], and need for cognition [60], the positive correlation between educational attainment and C-PETS scores was theoretically expected and empirically supported. These results suggest a positive association between educational attainment and creativity-related traits, implying that these characteristics could potentially be fostered through formal education and targeted interventions.

### Demographic influences and age-related differences

The significantly high scores of the 50–59 age group in BT and TT suggests minor variations in how different age groups perceive their own thinking styles. Given the very small effect sizes (*η*^2^ = 0.01), these results should be interpreted strictly as subtle cross-sectional differences in self-reported scores between cohorts.

Previous performance-based research indicates that divergent thinking capabilities, such as fluency and originality, typically peak in young adulthood (ages 20–35) and stabilize or decline thereafter [37]. However, in this study, the 50–59 age group demonstrated high scores in both BT, reflecting flexibility and a broad perspective, and TT, representing a systematic and thorough approach. This is consistent with the finding that indices of psychological functional integration peak between the ages of 55 and 60 [61]. While fluid intelligence typically declines from around age 20, experience-based competencies such as crystallized intelligence and emotional intelligence are maintained or enhanced [62]. Empirically, it has been shown that older adults (aged 64–78) are better able to avoid fixation on conventional solutions during the initial stages of a task and generate more original and expansive ideas than younger adults (aged 18–23) [61]. This advantage is attributed to the ability to intuitively use broad remote associations grounded in accumulated knowledge [61]. Furthermore, considering that convergent thinking is often fostered through formal education [28], the high BT and TT scores observed in this study underscore the maturation of these multifaceted associations and systematic verification processes. However, this study’s findings likely reflect subtle nuances in subjective perceptions of cognitive approaches rather than substantial shifts in actual creative capacity associated with aging, consistent with the minimal magnitude of the effect sizes.

No statistically significant gender differences were observed in BT, TT, IM, and the overall C-PETS scores. However, significant gender differences were observed in the sub-factors CS and AT. This finding aligns with the review [38], which suggests that overall creative capacity is relatively similar across genders, despite differences at the sub-factor level. This implies that understanding individual differences in creativity requires close attention to specific sub-factors rather than relying solely on global scores. The significantly higher scores of men in CS may be attributed to qualitative differences in achievement motivation formed through the socialization process. Men tend to exhibit higher achievement motivation in competitive contexts and the pursuit of excellence, which may be reflected in their propensity to tackle demanding goals [63]. Furthermore, CS is structured around motivational components such as need for cognition and curiosity. Although meta-analyses indicate no prominent gender differences in the personality of “Ideas” (a preference for speculation), men have been reported to surpass women in achievement motivation [64]. Because CS encompasses proactive attitudes, these qualitative differences in self-assertion and competitive motivation likely manifested in the self-reported scores.

Conversely, women scored significantly higher in AT. Because women are frequently required to integrate and process polysemous interpersonal signals and complex contextual information, they may possess a greater capacity to tolerate uncertainty and positively embrace diverse interpretations [65], which explains this finding. Additionally, the significantly higher AT scores observed in women may reflect an active cognitive style that avoids premature cognitive closure [66]. The ability to sustain thinking while tolerating the psychological discomfort inherent in uncertain situations without escaping into simplistic conclusions is consistent with the core characteristics of a low need for cognitive closure [66]. Thus, this finding may represent a form of cognitive persistence, characterized by the capacity to withhold judgment and keep exploring potential solutions despite incomplete information [66]. Notably, no gender difference was observed in the overall C-PETS score. This implies that although men and women may differ in their creative styles and the resources they use, their creative potential remains equivalent. Future research should investigate how these differences in self-perceived thinking styles translate into actual creative processes and achievements.

Regarding final educational attainment, differences in creativity were demonstrated. Previous evidence indicates that educational interventions such as problem-solving and scientific reasoning may significantly enhance students’ scientific creativity [67]. Particularly, problem-solving approaches have been reported to yield substantial effects. This suggests that programs systematically teaching cognitive skills, such as problem formulation and concept integration, are more effective than standard lectures. Furthermore, previous research [68] suggests that project-based learning education leads to back-and-forth between divergent and convergent thinking, as well as improved autonomy and motivation. In project-based learning, the creative process requires students to independently explore information, adjust multiple variables (constraints, costs, materials, etc.), and construct unique solutions to solve problems. This may enhance cognitive flexibility. While this study only examined educational attainment and did not specify the content of the education, prior research indicates that education can enhance creativity. The results of this study provide empirical support for the theoretical link between educational attainment and creative potential, consistent with the view that education plays a role in shaping creative traits.

Conversely, previous studies have explored how differences in educational content can influence the enhancement of creativity [69]. This study measured changes in creativity—as assessed by the TTCT—before and after education in the visual arts, music, and science, demonstrating that the degree of score improvement varied across domains. The participants were aged 15 to 17, and the focus was on the domain-specific effects of creativity education. While the present study examined differences related to the impact of higher education, further research is required to investigate differences related to various other academic fields.

### Limitations and future directions

The C-PETS was designed to measure multiple components of personality, thinking styles, and motivation as constituents of creativity. This scale does not assess intellectual ability, knowledge, or environmental factors, which are crucial components of creativity. Therefore, the high C-PETS scores do not necessarily guarantee creative outcomes because creativity involves the complex interaction of multiple factors beyond those measured using this scale. This exclusion was a deliberate methodological decision reflecting the constraints of self-report measurement rather than a theoretical oversight.

The initial item screening (167 to 42 items) was performed by the author to ensure theoretical consistency regarding domain-general components. Because this was a single-author project, inter-rater agreement (e.g., Cohen’s kappa) was not calculated. Although this differs from a multi-expert consensus approach, potential subjectivity was addressed through large-scale empirical validation in Study 1. Future research should focus on multilingual validation to confirm the scale’s applicability across diverse linguistic contexts.

Future research should investigate the relationships between the C-PETS and unmeasured components of creativity, such as intellectual ability, domain-specific knowledge, and environmental influences. In addition, conducting longitudinal studies to examine predictive validity for creative performance would further strengthen the scale’s empirical foundation.

Another limitation of this study is that measurement invariance across different age groups was not formally tested. While the findings indicate age-related differences in the mean scores of certain subscales, it remains unclear whether the underlying factor structure of the C-PETS is strictly equivalent across early and middle adulthood. Future research using multigroup CFA with larger samples is necessary to ensure the structural stability of the scale across early to middle adulthood. Furthermore, it is worth considering whether separate versions or developmental stage-specific norms should be established. Such investigations would clarify whether the observed differences reflect genuine age-related variations in creative potential or variations in the conceptualization of creativity-related traits at different stages of life.

## Conclusions

Despite this study’s limitations, the C-PETS represents a valuable contribution to creativity assessment by providing a multifaceted measure of creativity-related personality, thinking styles, and motivation. Directions for future research include developing interventions to enhance C-PETS scores and investigating the interactive effects of the C-PETS with intellectual ability, knowledge, and environmental factors on creative outcomes. Such research will advance understanding of the multifaceted nature of creativity and contribute to the development of evidence-based approaches to foster creative potential.

## Supplementary Information

Supplementary Material 1. C-PETS: The Original 167 Items. Description of data: This table provides a comprehensive item-level decision log for the original pool of 167 items. It includes each item’s category, underlying construct, original source reference, and the specific reason for its retention or exclusion during the qualitative screening process.

Supplementary Material 2. Item-level descriptive statistics and inter-item correlation matrix. Description of data: This spreadsheet provides the mean scores, standard deviations, and inter-item correlation matrix for the final 16 items of the C-PETS to ensure measurement stability and confirm the absence of item redundancy.

Supplementary Material 3. The 16 Items of the Creative Personality and Thinking Styles Scale. Description of data: This file contains the C-PETS instructions and question sequence that were used in the study.

## Data Availability

The datasets generated and analyzed in the current study are available in the OSF repository, [https://doi.org/10.17605/OSF.IO/4B92D].

## Abbreviations

AES: Aesthetic Experiences Scale
AT: Ambiguity Tolerance
BT: Broad Thinking
CFA: confirmatory factor analysis
C-PETS: Creative Personality and Thinking Styles Scale
CS: Challenge-Seeking
CSE: Creative Self-Efficacy
CPI: Creative Personal Identity
DAT: Divergent Association Task
EFA: exploratory factor analysis
IM: Information Manipulation
SSCS: Short Scale of Creative Self-Efficacy
TT: Thorough Thinking
TTCT: Torrance Tests of Creative Thinking
