# Supplementary material for: Development and validation of the creative personality and thinking styles scale: a multifaceted measure for assessing creativity-related individual differences
Source: BMC Psychol. 2026 Jun 8;14:1180. doi: 10.1186/s40359-026-04959-8 (PMC13465646; doi:10.1186/s40359-026-04959-8)
Supplement: Supplementary file 1 [file 40359_2026_4959_MOESM1_ESM.docx]

**Additional file 1**

**C-PETS: The Original 167 Items**

| No. | Item | Category | Construct | Exclusion reason or item no. | Reference |
| --- | --- | --- | --- | --- | --- |
| 1 | I am original, comes up with new ideas. | Openness to experience | Personality | Duplicate entries | [43] |
| 2 | I am ingenious, a deep thinker. | Openness to experience | Personality | Duplicate entries | [43] |
| 3 | I value inspiration. | Openness to experience | Personality | No.41 | [43] |
| 4 | I have high standards for my performance at work or at school. | Adaptive perfectionism | Personality | Duplicate entries | [44] |
| 5 | If you don't expect much out of yourself, you will never succeed. | Adaptive perfectionism | Personality | Outside the scope | [44] |
| 6 | People say I am hard on myself. | Adaptive perfectionism | Personality | No.23 | [44] |
| 7 | I set very high standards for myself. | Adaptive perfectionism | Personality | Duplicate entries | [44] |
| 8 | I expect the best from myself. | Adaptive perfectionism | Personality | Duplicate entries | [44] |
| 9 | I try to do my best at everything I do. | Adaptive perfectionism | Personality | Duplicate entries | [44] |
| 10 | I have a strong need to strive for excellence. | Adaptive perfectionism | Personality | Duplicate entries | [44] |
| 11 | If I do not set the highest standards for myself, I am likely to end up a second-rate person. | Adaptive perfectionism | Personality | Outside the scope | [44] |
| 12 | It is important to me that I be thoroughly competent in everything I do. | Adaptive perfectionism | Personality | Duplicate entries | [44] |
| 13 | It is important to set high standards for myself. | Adaptive perfectionism | Personality | No.25 | [44] |
| 14 | I am very good at focusing my efforts on attaining a goal. | Adaptive perfectionism | Personality | Multiple concepts | [44] |
| 15 | I hold the highest standards. | Adaptive perfectionism | Personality | No.24 | [44] |
| 16 | Other people seem to accept lower standards from themselves than I do. | Adaptive perfectionism | Personality | Outside the scope | [44] |
| 17 | I expect higher performance in my daily tasks than most people. | Adaptive perfectionism | Personality | Multiple concepts | [44] |
| 18 | I decline invitations that are not beneficial for me. | Adaptive perfectionism | Personality | No.22 | [44] |
| 19 | I take the time to consider multiple ways to solve a problem, | cognitive flexibility | Thinking styles | Duplicate entries | [25] |
| 20 | When thinking about things, I try to understand the bigger picture. | cognitive flexibility | Thinking styles | No.33 | [25] |
| 21 | I can quickly direct my focus to a single task when needed. | cognitive flexibility | Thinking styles | No.40 | [25] |
| 22 | I would prefer to consider several ways to tackle a problem. | cognitive flexibility | Thinking styles | No.35 | [25] |
| 23 | I can work on many things at once. | cognitive flexibility | Thinking styles | No.38 | [25] |
| 24 | I review my work for mistakes. | cognitive flexibility | Thinking styles | No.37 | [25] |
| 25 | I am able to quickly find the right way on the spot, | cognitive flexibility | Thinking styles | Outside the scope | [25] |
| 26 | I am willing to listen and consider alternatives for solving problems. | cognitive flexibility | Thinking styles | No.34 | [25] |
| 27 | I have a diverse range of ideas. | cognitive flexibility | Thinking styles | No.36 | [25] |
| 28 | I look at situations objectively to solve problems, | cognitive flexibility | Thinking styles | Duplicate entries | [25] |
| 29 | I am able to adopt new ideas, | cognitive flexibility | Thinking styles | Duplicate entries | [25] |
| 30 | I can shift my focus between two different tasks, | cognitive flexibility | Thinking styles | No.39 | [25] |
| 31 | I don't tolerate ambiguous situations well. | Ambiguity tolerance | Personality | Duplicate entries | [14] |
| 32 | I find it difficult to respond when faced with an unexpected event. | Ambiguity tolerance | Personality | Outside the scope | [14] |
| 33 | I don't think new situations are any more threatening than familiar situations. | Ambiguity tolerance | Personality | Outside the scope | [14] |
| 34 | I'm drawn to situations which can be interpreted in more than one way. | Ambiguity tolerance | Personality | Duplicate entries | [14] |
| 35 | I would rather avoid solving a problem that must be viewed from several different perspectives. | Ambiguity tolerance | Personality | Multiple concepts | [14] |
| 36 | I try to avoid situations which are ambiguous. | Ambiguity tolerance | Personality | Duplicate entries | [14] |
| 37 | I am good at managing unpredictable situations. | Ambiguity tolerance | Personality | Duplicate entries | [14] |
| 38 | It is interesting to interpret things in many different ways. | Ambiguity tolerance | Personality | No.29 | [14] |
| 39 | Problems which cannot be considered from just one point of view are a little threatening. | Ambiguity tolerance | Personality | Outside the scope | [14] |
| 40 | I avoid situations which are too complicated for me to easily understand. | Ambiguity tolerance | Personality | Duplicate entries | [14] |
| 41 | To some extent, I am able to accept imperfections. | Ambiguity tolerance | Personality | No.31 | [14] |
| 42 | My imagination is sparked by the unfamiliar. | Ambiguity tolerance | Personality | No.28 | [14] |
| 43 | I try to avoid problems which don't seem to have only one "best" solution. | Ambiguity tolerance | Personality | Multiple concepts | [14] |
| 44 | I often find myself looking for something new, rather than trying to hold things constant in my life. | Ambiguity tolerance | Personality | Outside the scope | [14] |
| 45 | I generally prefer novelty over familiarity. | Ambiguity tolerance | Personality | Outside the scope | [14] |
| 46 | I dislike ambiguous situations. | Ambiguity tolerance | Personality | Duplicate entries | [14] |
| 47 | Some problems are so complex that just trying to understand them is fun. | Ambiguity tolerance | Personality | Duplicate entries | [14] |
| 48 | I have little trouble coping with unexpected events. | Ambiguity tolerance | Personality | Duplicate entries | [14] |
| 49 | I pursue problem situations which are so complex some people call them "mind boggling". | Ambiguity tolerance | Personality | Duplicate entries | [14] |
| 50 | I find it hard to make a choice when the outcome is uncertain. | Ambiguity tolerance | Personality | Outside the scope | [14] |
| 51 | I enjoy an occasional surprise. | Ambiguity tolerance | Personality | Duplicate entries | [14] |
| 52 | Imperfections are interesting. | Ambiguity tolerance | Personality | No.30 | [14] |
| 53 | I have often been able to come up with a new idea myself based on an idea from someone else. | Divergent thinking | Thinking styles | Duplicate entries | [15, 45] |
| 54 | Creative people generally seem to have scrambled minds. | Divergent thinking | Thinking styles | Outside the scope | [15, 45] |
| 55 | Ideas are fundamental to decision making, and as such should not be taken for granted. | Divergent thinking | Thinking styles | Outside the scope | [15, 45] |
| 56 | New ideas seldom work out. | Divergent thinking | Thinking styles | Outside the scope | [15, 45] |
| 57 | Most people never really "open their minds" to all the possible options or alternatives when considering solutions to a problem. | Divergent thinking | Thinking styles | Duplicate entries | [15, 45] |
| 58 | Truly creative people also have unusual lifestyles. | Divergent thinking | Thinking styles | Outside the scope | [15, 45] |
| 59 | In organizations, senior management should encourage ideas by demonstrating, they are willing to act on them. | Divergent thinking | Thinking styles | Multiple concepts | [15, 45] |
| 60 | I'm often too embarrassed to suggest my wild ideas. People laugh and suspect I can't think straight. | Divergent thinking | Thinking styles | Multiple concepts | [15, 45] |
| 61 | I really enjoy the challenge of finding a different way to solve a problem. | Divergent thinking | Thinking styles | Duplicate entries | [15, 45] |
| 62 | Really creative people are never very organized. | Divergent thinking | Thinking styles | Outside the scope | [15, 45] |
| 63 | Some people really surprise me with their new ideas. | Divergent thinking | Thinking styles | Outside the scope | [15, 45] |
| 64 | I don't have much time for thinking up wild ideas, I'm too busy just getting my job done. | Divergent thinking | Thinking styles | Outside the scope | [15, 45] |
| 65 | Most innovations occur because of needs or problems. | Divergent thinking | Thinking styles | Outside the scope | [15, 45] |
| 66 | Why is everybody always talking about ideas? I've got more work now than I know what to do with. | Divergent thinking | Thinking styles | Multiple concepts | [15, 45] |
| 67 | When I get a new idea, I really get excited. | Divergent thinking | Thinking styles | Duplicate entries | [15, 45] |
| 68 | Ideas are only important if they impact on major projects. | Divergent thinking | Thinking styles | Outside the scope | [15, 45] |
| 69 | All people have creative ideas from time to time. | Divergent thinking | Thinking styles | Outside the scope | [15, 45] |
| 70 | The boss' idea is usually always the best since it comes from a much broader perspective. | Divergent thinking | Thinking styles | Multiple concepts | [15, 45] |
| 71 | Crazy sounding ideas can lead to something. | Divergent thinking | Thinking styles | Outside the scope | [15, 45] |
| 72 | Listening to other people's ideas is a waste of time. | Divergent thinking | Thinking styles | Duplicate entries | [15, 45] |
| 73 | Productive change is important to a business. New ideas foster change. Therefore, new ideas are important to business. | Divergent thinking | Thinking styles | Ambiguous meaning | [15, 45] |
| 74 | If everyone is providing ideas, then no one gets any work done. | Divergent thinking | Thinking styles | Outside the scope | [15, 45] |
| 75 | The more problems I have, the more opportunities I have. | Divergent thinking | Thinking styles | Multiple concepts | [15, 45] |
| 76 | Only smart, knowledgeable people have good ideas. | Divergent thinking | Thinking styles | Multiple concepts | [15, 45] |
| 77 | I have many rough ideas. | Divergent thinking | Thinking styles | No.1 | [15, 45] |
| 78 | I think about ideas more often than most people. | Divergent thinking | Thinking styles | Outside the scope | [15, 45] |
| 79 | When I come up with a new idea, I get really excited. | Divergent thinking | Thinking styles | No.4 | [15, 45] |
| 80 | I can convert an object with a specific purpose into one with a different function. | Divergent thinking | Thinking styles | No.42 | [15, 45] |
| 81 | I come up with an idea or solution other people  have never thought of. | Divergent thinking | Thinking styles | Duplicate entries | [15, 45] |
| 82 | I like to play around with ideas for the fun of it. | Divergent thinking | Thinking styles | Multiple concepts | [15, 45] |
| 83 | It is important to be able to think of bizarre and  wild possibilities. | Divergent thinking | Thinking styles | Ambiguous meaning | [15, 45] |
| 84 | I trust my creativity. | Divergent thinking | Thinking styles | No.32 | [15, 45] |
| 85 | I have always been an active thinker—I have  lots of ideas. | Divergent thinking | Thinking styles | Outside the scope | [15, 45] |
| 86 | I enjoy having leeway in the things I do and  room to make up my own mind. | Divergent thinking | Thinking styles | Outside the scope | [15, 45] |
| 87 | I would take a college course which was based  on original ideas. | Divergent thinking | Thinking styles | Outside the scope | [15, 45] |
| 88 | I am able to think about things intensely for  many hours. | Divergent thinking | Thinking styles | Duplicate entries | [15, 45] |
| 89 | I try to exercise my mind by thinking things  through. | Divergent thinking | Thinking styles | Outside the scope | [15, 45] |
| 90 | I am able to think up answers to problems that  haven’t already been figured out. | Divergent thinking | Thinking styles | Outside the scope | [15, 45] |
| 91 | I am good at combining ideas in ways that others have not tried before. | Divergent thinking | Thinking styles | No.2 | [15, 45] |
| 92 | Friends ask me to help them think of ideas and  solutions. | Divergent thinking | Thinking styles | Multiple concepts | [15, 45] |
| 93 | I have ideas about new inventions or about how  to improve things. | Divergent thinking | Thinking styles | Duplicate entries | [15, 45] |
| 94 | My ideas are often considered “impractical” or even “wild.” | Divergent thinking | Thinking styles | Outside the scope | [15, 45] |
| 95 | Sometimes I get so interested in a new idea that I forget about other things that I should be doing. | Divergent thinking | Thinking styles | Duplicate entries | [15, 45] |
| 96 | I often have trouble sleeping at night, because  so many ideas keep popping into my head. | Divergent thinking | Thinking styles | Multiple concepts | [15, 45] |
| 97 | When writing papers or talking to people, I often have trouble staying with one topic because  I think of so many things to write or say. | Divergent thinking | Thinking styles | Multiple concepts | [15, 45] |
| 98 | I come up with new ideas by building on others’ ideas. | Divergent thinking | Thinking styles | No.3 | [15, 45] |
| 99 | Some people might think me scatterbrained or  absentminded because I think about a variety of  things at once. | Divergent thinking | Thinking styles | Multiple concepts | [15, 45] |
| 100 | When I meet with a new thing, I want to learn as much as possible about it. | Absorption | Personality | Duplicate entries | [47] |
| 101 | I tend to become absorbed in one thing. | Absorption | Personality | No.26 | [47] |
| 102 | I often try to learn something new and ask many questions. | Absorption | Personality | Duplicate entries | [47] |
| 103 | When I am interested in something, it takes time to interrupt me. | Absorption | Personality | Ambiguous meaning | [47] |
| 104 | When I become interested in something, I become completely absorbed in it. | Absorption | Personality | No.27 | [47] |
| 105 | Everywhere I go I am searching for new things to do or see. | Absorption | Personality | Outside the scope | [47] |
| 106 | New ideas excite my imagination. | Curiosity | Personality | Duplicate entries | [47] |
| 107 | I like to take on new challenges. | Curiosity | Personality | No.13 | [47] |
| 108 | Learning about subjects which are unfamiliar. | Curiosity | Personality | Duplicate entries | [47] |
| 109 | Fascinating to learn new information. | Curiosity | Personality | Duplicate entries | [47] |
| 110 | Thinking over new ideas and concepts is fun. | Curiosity | Personality | Duplicate entries | [47] |
| 111 | Find myself turning new ideas over/think about them. | Curiosity | Personality | Outside the scope | [47] |
| 112 | I have an interest in many things. | Curiosity | Personality | No.15 | [47] |
| 113 | Discussing theories with a philosopher. | Curiosity | Personality | Ambiguous meaning | [47] |
| 114 | When I learn new things, I want to research them thoroughly. | Curiosity | Personality | No.17 | [47] |
| 115 | Find it interesting to think about contradicting ideas. | Curiosity | Personality | Outside the scope | [47] |
| 116 | Discussing abstract concepts. | Curiosity | Personality | Outside the scope | [47] |
| 117 | Hypothetical situation/think about what might happen. | Curiosity | Personality | Outside the scope | [47] |
| 118 | Thinking about different answers to same question. | Curiosity | Personality | Outside the scope | [47] |
| 119 | Someone answers a question/even more inquisitive. | Curiosity | Personality | Outside the scope | [47] |
| 120 | Figure out what led up to important historical events. | Curiosity | Personality | Outside the scope | [47] |
| 121 | Theory about something/like to test it out. | Curiosity | Personality | Outside the scope | [47] |
| 122 | I am very interested in doing things that no one has done before. | Curiosity | Personality | No.14 | [47] |
| 123 | See a word I don’t know/like to look up the meaning. | Curiosity | Personality | Duplicate entries | [47] |
| 124 | Complicated piece of machinery/ask how it works. | Curiosity | Personality | Outside the scope | [47] |
| 125 | Arithmetic problem/enjoy imagining solutions. | Curiosity | Personality | Outside the scope | [47] |
| 126 | Like to understand how complicated things work. | Curiosity | Personality | Outside the scope | [47] |
| 127 | I keep thinking until I reach a clear and precise answer. | Curiosity | Personality | No.16 | [47] |
| 128 | Interested in discovering how things work. | Curiosity | Personality | Duplicate entries | [47] |
| 129 | Complex problem/interested in discovering a solution. | Curiosity | Personality | Duplicate entries | [47] |
| 130 | Riddle/interested in trying to solve it. | Curiosity | Personality | Outside the scope | [47] |
| 131 | Simple explanations leave a lot of questions. | Curiosity | Personality | Outside the scope | [47] |
| 132 | Read magazine that reports new scientific discoveries. | Curiosity | Personality | Outside the scope | [47] |
| 133 | When an unexpected event occurs, I investigate it until I determine the cause. | Curiosity | Personality | No.18 | [47] |
| 134 | I prefer complex problems to simple ones. | Need for cognition | Motivation | No.12 | [22] |
| 135 | I like to have the responsibility of handling a situation that requires a lot of thinking. | Need for cognition | Motivation | Outside the scope | [22] |
| 136 | Thinking is not my idea of fun. | Need for cognition | Motivation | Outside the scope | [22] |
| 137 | I research unclear information until I fully understand it. | Need for cognition | Motivation | No.11 | [22] |
| 138 | I try to anticipate and avoid situations where there is likely chance I will have to think in depth  about something. | Need for cognition | Motivation | Outside the scope | [22] |
| 139 | I find satisfaction in deliberating hard and for long hours. | Need for cognition | Motivation | Duplicate entries | [22] |
| 140 | I only think as hard as I have to. | Need for cognition | Motivation | Outside the scope | [22] |
| 141 | I prefer to think about small, daily projects to long-term ones. | Need for cognition | Motivation | Duplicate entries | [22] |
| 142 | I like tasks that require little thought once I've learned them. | Need for cognition | Motivation | Outside the scope | [22] |
| 143 | The idea of relying on thought to make my way to the top appeals to me. | Need for cognition | Motivation | Outside the scope | [22] |
| 144 | I really enjoy a task that involves coming up with new solutions to problems. | Need for cognition | Motivation | Duplicate entries | [22] |
| 145 | Learning new ways to think doesn't excite me very much. | Need for cognition | Motivation | Duplicate entries | [22] |
| 146 | I prefer my life to be filled with puzzles that I must solve. | Need for cognition | Motivation | Outside the scope | [22] |
| 147 | The notion of thinking abstractly is appealing to me. | Need for cognition | Motivation | Outside the scope | [22] |
| 148 | I would prefer a task that is intellectual. difficult. and important to one that is somewhat important but does not require much thought. | Need for cognition | Motivation | Outside the scope | [22] |
| 149 | I feel relief rather than satisfaction after completing a task that required a lot of mental effort. | Need for cognition | Motivation | Multiple concepts | [22] |
| 150 | It's enough for me that something gets the job done; I don't care how or why it works. | Need for cognition | Motivation | Duplicate entries | [22] |
| 151 | I usually end up deliberating about issues even when they do not affect me personally | Need for cognition | Motivation | Outside the scope | [22] |
| 152 | I reflect on important information as I come across it. | Metacognitive Awareness | Thinking Styles | No.5 | [46] |
| 153 | I consciously direct my attention toward important information. | Metacognitive Awareness | Thinking Styles | No.6 | [46] |
| 154 | I provide my own examples to make information more meaningful. | Metacognitive Awareness | Thinking Styles | No.7 | [46] |
| 155 | I draw diagrams to enhance my understanding. | Metacognitive Awareness | Thinking Styles | No.8 | [46] |
| 156 | I connect new information to what I already know. | Metacognitive Awareness | Thinking Styles | No.9 | [46] |
| 157 | I focus on the overall meaning rather than on specific details. | Metacognitive Awareness | Thinking Styles | No.10 | [46] |
| 158 | I ask myself if what I’m reading is related to what I already know. | Metacognitive Awareness | Thinking Styles | Duplicate entries | [46] |
| 159 | I try to break studying down into smaller steps. | Metacognitive Awareness | Thinking Styles | Outside the scope | [46] |
| 160 | I find my thoughts wandering spontaneously. | Mind-wandering | Thinking Styles | Duplicate entries | [48] |
| 161 | When my mind wanders, it tends to jump from one to another. | Mind-wandering | Thinking Styles | No.21 | [48] |
| 162 | I let my mind wander even when I am supposed to be doing something else. | Mind-wandering | Thinking Styles | No.20 | [48] |
| 163 | I let my thoughts wander on purpose. | Mind-wandering | Thinking Styles | No.19 | [48] |
| 164 | I enjoy mind-wandering. | Mind-wandering | Thinking Styles | Ambiguous meaning | [48] |
| 165 | I find mind-wandering is a good way to cope with boredom. | Mind-wandering | Thinking Styles | Ambiguous meaning | [48] |
| 166 | I allow myself to get absorbed in pleasant fantasy. | Mind-wandering | Thinking Styles | Outside the scope | [48] |
| 167 | It feels like I don’t have control over when my mind wanders. | Mind-wandering | Thinking Styles | Outside the scope | [48] |
