# Supplementary material for: Development and validation of the creative personality and thinking styles scale: a multifaceted measure for assessing creativity-related individual differences
Source: BMC Psychol. 2026 Jun 8;14:1180. doi: 10.1186/s40359-026-04959-8 (PMC13465646; doi:10.1186/s40359-026-04959-8)
Supplement: Supplementary file 2 [file 40359_2026_4959_MOESM3_ESM.docx]

**Additional file 3**

**The 16 items of the Creative Personality and Thinking Styles Scale**

For each of the following statements, please indicate the extent to which it applies to you.

| Strongly  disagree | Disagree | Neither yes or no | Agree | Strongly agree |
| --- | --- | --- | --- | --- |
| 1 | 2 | 3 | 4 | 5 |
| 1. I am very interested in doing things that no one has done before. | | | | |
| 2. I keep thinking until I reach a clear and precise answer. | | | | |
| 3. Imperfections are interesting. | | | | |
| 4. I connect new information to what I already know. | | | | |
| 5. I like to take on new challenges. | | | | |
| 6. I am willing to listen and consider alternatives for solving problems. | | | | |
| 7. To some extent, I am able to accept imperfections. | | | | |
| 8. When an unexpected event occurs, I investigate it until I determine the cause. | | | | |
| 9. I provide my own examples to make information more meaningful. | | | | |
| 10. When thinking about things, I try to understand the bigger picture. | | | | |
| 11. When I learn new things, I want to research them thoroughly. | | | | |
| 12. It is interesting to interpret things in many different ways. | | | | |
| 13. I have a diverse range of ideas. | | | | |
| 14. I would prefer to consider several ways to tackle a problem. | | | | |
| 15. I reflect on important information as I come across it. | | | | |
| 16. I prefer complex problems to simple ones. | | | | |
